# Serial Interferon-Gamma Release Assay (IGRA) Testing to Monitor Treatment Responses in Cases of Feline Mycobacteriosis

**DOI:** 10.3390/pathogens10060657

**Published:** 2021-05-26

**Authors:** Jordan L. Mitchell, Conor O’Halloran, Paul Stanley, Kieran McDonald, Paul Burr, Danièlle A. Gunn-Moore, Jayne C. Hope

**Affiliations:** 1 The Royal (Dick) School of Veterinary Studies and The Roslin Institute, Easter Bush Campus, University of Edinburgh, Midlothian EH25 9RG, UKpaulstanley20@hotmail.co.uk (P.S.); danielle.gunn-moore@ed.ac.uk (D.A.G.-M.); jayne.hope@roslin.ed.ac.uk (J.C.H.); 2 Biobest Laboratories Ltd., Edinburgh Technopole, Milton Bridge EH26 0PY, UK; kieran.mcdonald@biobest.co.uk (K.M.); paul.burr@biobest.co.uk (P.B.)

**Keywords:** cat, tuberculosis, mycobacteria, monitoring, interferon-gamma release assay, diagnostics

## Abstract

The interferon-gamma release assay (IGRA) is used to diagnose cases of feline mycobacteriosis, but the use of serial testing to monitor treatment responses has not been evaluated in this species. From a population of cats that underwent IGRA testing for diagnostic investigation, individuals were identified with a pre- and end-of-treatment IGRA that passed control thresholds. The number of cats which reverted to negative at the end-of-treatment IGRA, changes in paired antigen-specific optical density (OD) values and differences in the pre-treatment antigen-specific OD values for those which underwent reversion were compared. Factors to explain reversion or recurrence of disease post-treatment were explored. Four of 18 cats (22%) reverted to negativity at the point of clinical resolution (*p* = 0.33), there was no difference in paired antigen-specific OD values (*p* ≥ 0.12), and cats that reverted did not have a lower baseline OD value (*p* = 0.63). No statistically significant factors were identified to predict reversion (*p* ≥ 0.08). Remaining positive at the end of treatment IGRA was not associated with recurrence of disease post-treatment (*p* = 0.34). Overall, these data suggest there is limited value in the use of the IGRA to monitor treatment responses in cats.

## 1. Introduction

Mycobacteriosis is a substantial issue within the domestic cat population in Great Britain, with an estimated 1% of all routine feline biopsy submissions showing changes suggestive of mycobacterial disease [1]; however, many cases may not initially be recognised by practitioners. There are many challenges when faced with a suspected case of mycobacterial disease, notably obtaining an accurate diagnosis, monitoring the response to therapy, and identifying when treatment with antimicrobials can be stopped. Current options for diagnosing mycobacterial infections in cats are limited to specialist mycobacterial culture, molecular-based methods, i.e., polymerase chain reaction (PCR) and genome sequencing, or the interferon-gamma (IFNγ) release assay (IGRA) [2].

Initially established for diagnosing tuberculosis (TB) in cattle where tuberculin skin testing failed to identify all infected animals [3], the IGRA has since been adapted for the diagnosis of TB in other species [4,5], including cats [6]. These assays detect the cellular immune response through secretion of IFNγ by T-cells following stimulation with mycobacterial antigens [7]. The feline IGRA is designed to detect and discriminate between infection with the two most common causes of mycobacteriosis in this species in Great Britain, i.e., *Mycobacterium* (*M.*) *microti* and *M. bovis* [8], which both cause TB. The IGRA also has some capacity to detect infections with *M. avium* and other non-tuberculous mycobacteria [9]. While the IGRA appears useful for diagnosing mycobacteriosis in cats, its clinical utility for monitoring the response to treatment in this species is unknown.

The IGRA is an immunological test based on sensitised antigen-specific T-cells responding to repeated antigenic stimulation in vitro [7], and its methodologies and use in people have been thoroughly evaluated [10]. It is thought that the number of IFNγ-secreting effector T-cells is proportional to the antigenic load, which is taken to reflect the bacterial load [11,12], and that successful antimycobacterial treatment will result in a decrease of this antigenic stimulus and a subsequent reduction in the number of antigenic-specific effector T-cells [13]. Therefore, it has been proposed that serial IGRA testing for those receiving antimycobacterial chemotherapy may be of some benefit [14]. Early investigations of serial IFNγ responses showed that region of difference-1 (RD-1) markers, such as early secreted antigenic target 6kDa (ESAT-6), were more likely to decrease in those with a good response to treatment than responses to the crude purified protein derivative (PPD) antigen [15], and human IGRA tests have since been designed using RD-1 antigens. Despite this, in people, reversion from IGRA positivity at the time of diagnosis to negativity at the conclusion of therapy has been shown to range from as low as 5.5% [16] up to 71% [17]. However, remaining positive at the end of treatment IGRA is not always associated with an unfavourable outcome [16,18]. While changes in the categorical classification of IGRA results has not been suggested as helpful for assessing successful response to therapy, the magnitude of change in the IFNγ response may be of more benefit, as a small majority of studies has shown a significant decrease in this measure between the start and end of treatment [19]. Despite this, some studies have shown no change between pre- and post-treatment IFNγ levels [20,21], while others have shown an increase [18]. There are limitations to some of the methodologies of these studies making it difficult to accurately compare and collate findings, namely, variable sample sizes, genetic differences across populations studied, the level of TB exposure across different populations, co-morbidities, and the censoring of data exceeding the upper limit of quantification. A systematic review of 30 studies using serial IGRA testing to monitor the response to anti-TB chemotherapy failed to show a consensus in the rate of reversion with highly variable results between patients within studies, as well as rates across studies. Therefore, repeat IGRA testing is not recommended to monitor individual patient responses to treatment in humans [19]. 

Serial IGRA testing has been previously reported in a small number of cats treated for confirmed or suspected mycobacteriosis [9,22], most of whom remained positive despite apparent resolution of disease. However, it is unknown whether these findings are representative of the IFNγ response in a larger population of cats or whether we should aim for reversion to IGRA negativity when treating cases of feline mycobacterial disease.

The objective of this study was to determine whether serial IGRA tests are of benefit for monitoring the response to antimycobacterial therapy in cats. We assessed whether reversion to IGRA negativity was observed in association with apparent clinical resolution. We also measured the change in the magnitude of the IFNγ response and investigated factors that might influence reversion to IGRA negativity or the risk of recurrence of mycobacterial disease post-treatment. 

## 2. Results

### 2.1. Study Population Selection and Summary Statistics

**Figure 1:**
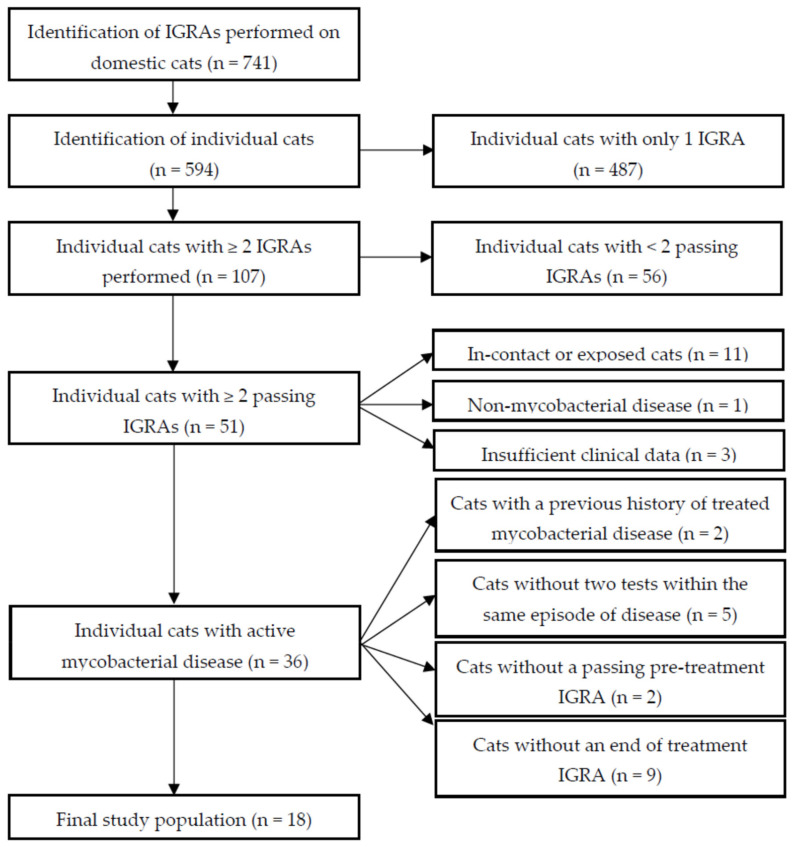
Flow diagram to show the decision-making process for identifying cats assessed by serial interferon-gamma release assay (IGRA) for subsequent analysis. IGRA = interferon-gamma release assay; Passing = optical density (OD) positive control (PC) ≥ 0.40, OD negative control (NC) ≤ 0.30, and OD_PC_-OD_NC_ ≥ 0.10, or if OD_PC_ < 0.40, OD_PC_-OD_NC_ ≥ 0.20. Coefficient of variation between duplicate PC and NC wells ≤ 30%.

The selection process to identify eligible cats that underwent serial IGRA testing while receiving antimycobacterial treatment is shown in Figure 1. In total, 741 IGRAs were performed on 594 individual cats, with 107 cats having had more than one test. Fifty-one cats had at least two tests which passed the test criteria (see *4.1. Study Population Selection*). Of the 56 cats that did not pass these criteria: 49 were excluded as they did not meet the conditions for a passing optical density (OD) positive control (PC) and/or positive control–negative control (NC) OD values, four had coefficient of variation (CV) values exceeding 30% for at least one test condition (including six that also did not meet the OD_PC_/OD_PC_-OD_NC_ thresholds), and three had average OD_NC_ values greater than 0.30. Active mycobacterial disease was diagnosed in 36 of the 51 cats with at least two tests meeting the IGRA inclusion criteria, and of these 18 were included for final analysis as they did not have a previous history of being treated for mycobacterial disease. Both tests passed quality control (QC) thresholds and they were conducted (pre-treatment and at the end of treatment) within the same episode of active clinical disease.

Clinical data for the cats in this study are summarised in Table 1. The median age of cats at the time of the pre-treatment IGRA was 6.75 years (0.5–13 years), and 15 (83%) were neutered males. Twelve cats (67%) were domestic short-hair (DSH); the remaining breeds were Siamese (n = 4), Bengal (n = 1), and Tonkinese (n = 1). Three cats were diagnosed with *M. bovis* by culture, one cat was culture- and PCR-positive for *M. microti,* and three further cats were diagnosed with *M. tuberculosis*-complex (MTBC) infection on PCR [23], but there was insufficient DNA to define the infectious agent further. Two cats were negative for *Mycobacterium* species on culture, and another cat was culture- and PCR-negative. The remaining eight cats did not undergo culture or PCR testing. Lymphadenopathy was identified in 13 cats (72%), including one cat with tonsillar lymphoid hyperplasia, followed by cutaneous lesions in seven (39%) and ocular lesions in three (17%). Twelve cats (67%) had an abnormal pulmonic lung pattern on radiography or computed tomography (CT). Testing for feline leukaemia virus antigen and feline immunodeficiency virus antibody was performed in five cats and all were negative. Hypercalcaemia was identified in four of nine cats tested (44%); one cat had increased total calcium, but ionised calcium was within the reference interval, another cat with increased total calcium did not have ionised calcium measured, and the remaining two cats both had increased ionised calcium.

Therapeutic interventions varied. The median duration of antimycobacterial treatment was six months (range 3–24 months). Ten cats (56%) were maintained on ‘triple therapy’ with rifampicin, azithromycin, or clarithromycin, and pradofloxacin for the entire duration of their treatment protocol; two of these cats also underwent surgery (i.e., enucleation) prior to medical management. Four cats (22%) were started on ‘triple therapy’, with discontinuation of rifampicin after two and six months in two cats, whereas azithromycin was discontinued in two cats after two and three months. Azithromycin or clarithromycin were substituted for erythromycin in one cat, in conjunction with rifampicin and pradofloxacin. One cat was treated with rifampicin, azithromycin, and marbofloxacin, with discontinuation of rifampicin after three months. A protocol of rifampicin, pradofloxacin, and clindamycin was started in one cat with suspected concurrent toxoplasmosis; clindamycin was then replaced with azithromycin after three months. The final cat was treated with a combination of erythromycin, doxycycline, and pradofloxacin.

Recurrence of clinical signs attributable to mycobacteriosis following resolution of disease was identified in five cases, all of which had radiographic evidence of pulmonic disease. The median duration to the onset of recurrent clinical signs was 16 months (3–27 months). Two of these cats were subsequently euthanased, whereas treatment was re-instated for the other three cats; clinical resolution was achieved in two of these cats, while the third was lost to follow-up.

### 2.2. Qualitative Classification of IGRA Results

Pre- and end-of-treatment IGRA results were interpreted according to the pattern of responses to each antigen and scored on a positive–negative basis (Table 2). All 18 cats were positive on the pre-treatment IGRA, showing a biased response to PPD from *M. bovis* (PPDB), suggesting infection with a member of the MTBC. Three cases were positive to early secreted antigenic target 6kDa-culture filtrate protein 10kDa (ESAT-6/CFP-10 [E6C10]); two were culture-positive for *M. bovis* (cases 9 and 11). One cat (case 2) had a culture-confirmed diagnosis of *M. bovis* infection, but was negative to E6C10 on IGRA. At the end-of-treatment IGRA, 14 (78%) were persistently positive and four (22%) underwent reversion to negativity. A PPDB-biased response was maintained in all 14 persistently positive cats. In addition to the two E6C10-positive cats with culture-confirmed *M. bovis* infection on the initial IGRA, two further cats were now positive to E6C10 at the end-of-treatment IGRA. Neither of these cats were case 2, the *M. bovis* culture-positive cat, which remained negative to E6C10. The proportion of cats that reverted to a negative end-of-treatment IGRA was not statistically different to that seen in humans treated for active TB (*p* = 0.33), i.e., the rates of reversion are similar. 

### 2.3. Quantitative Evaluation of IGRA Responses

To account for intra-cat variability between IGRA results that passed QC thresholds, paired OD_PC_ values for each cat at pre- and end-of-treatment IGRA were compared to identify overlap in these values, allowing for comparison of antigen OD values. Eleven of the 18 cats had overlapping OD_PC_ values between the pre- and end-of-treatment IGRA (data not shown).

**Figure 2:**
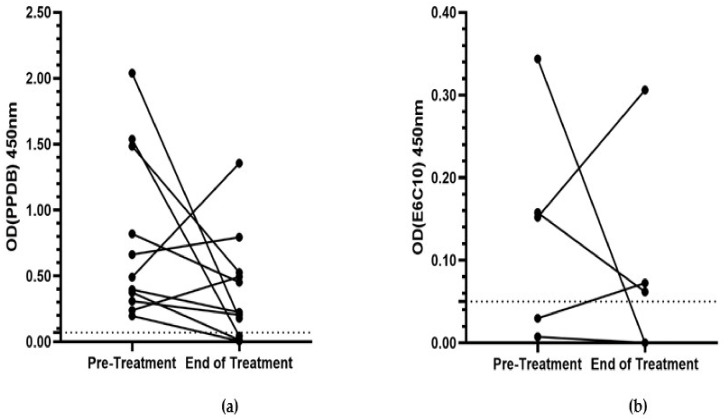
Paired average negative control corrected-OD values for pre- and end-of-treatment IGRA for (**a**) PPDB (n = 11) and (**b**) E6C10 (n = 5). The dotted line at OD 0.07 signifies the threshold for PPDB positivity. The dotted line at OD 0.05 signifies the threshold for E6C10 positivity. OD = optical density. IGRA = interferon-gamma release assay. PPDB = purified protein derivative from *M. bovis*. E6C10 = ESAT-6/CFP-10 antigenic cocktail.

Of these 11 cats, eight (73%) showed a decrease in the OD_PPDB_ value between the pre- and end-of-treatment IGRA (Figure 2A). There was a decrease in the OD_E6C10_ value in three of the five cats that were culture- or IGRA-positive for infection with *M. bovis* (Figure 2B). The median OD values for both PPDB and E6C10 decreased (0.49 to 0.22 OD_PPDB_; 0.15 to 0.06 OD_E6C10_), but there was no statistically significant difference between paired OD values for either antigen (OD_PPDB_, *p* = 0.12; OD_E6C10_, *p* = 0.81).

**Figure 3:**
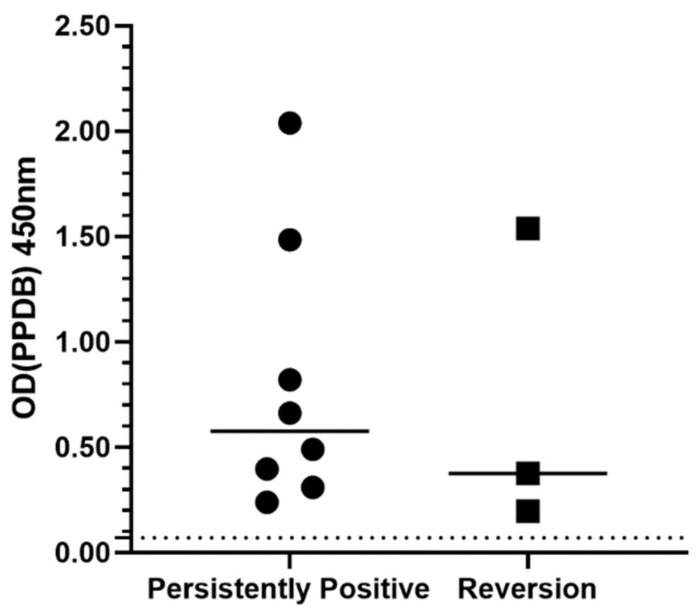
Pre-treatment IGRA average OD_PPDB_ values for cats who remained positive at the end-of-treatment IGRA (n = 8) compared to those which reverted to negative (n = 3). The solid line shows the median OD value for each group. The dotted line at OD 0.07 signifies the threshold for PPDB positivity. OD = optical density. IGRA = interferon-gamma release assay. PPDB = purified protein derivative from *M. bovis*.

The pre-treatment OD_PPDB_ values were compared between cats that were persistently positive at the end-of-treatment IGRA (n = 8) and those that reverted to negative (n = 3) (Figure 3). The median OD_PPDB_ value was higher in the persistently positive group (0.58) compared to the reversion group (0.38), however this difference was not statistically significant (*p* = 0.63). 

### 2.4. Logistic Regression Analysis

Logistic regression was performed to identify potential factors that may predict reversion to IGRA negativity at the end-of-treatment IGRA or recurrence of disease. For reversion, a multivariate model with age, gender, breed (DSH vs non-DSH), the presence or absence of pulmonic disease, and treatment regimen (‘triple therapy’ as at least part of the treatment protocol for at least three months in cases without pulmonic disease and at least six months in cases with pulmonic disease vs ‘triple therapy’ as at least part of the treatment protocol for an insufficient duration of time as well as all other protocols) was constructed. The multivariate model then underwent stepwise multidirectional reduction to its simplest components. Exploration of the full multivariate model revealed high collinearity between non-DSH cats and the presence of pulmonic disease, with no statistically significant factors for IGRA reversion identified. The reduced model identified younger age and the absence of pulmonic disease as the most important factors in predicting reversion to IGRA negativity at the end of treatment, but neither were statistically significant (age, *p* = 0.08, odds ratio [OR] = 0.50 [0.23−1.09]; pulmonic disease, *p* = 0.17, OR = 0.05 [0.0006–3.88]).

Factors included in the multivariate model for the risk of disease recurrence included those modelled for reversion, in addition to remaining persistently positive at the end-of-treatment IGRA. There was no statistically significant association between remaining persistently positive at the end-of-treatment IGRA and recurrence of disease (*p* = 0.34, OR = 0.06 [0.0002–19.26]). Quasi-complete separation of the data was identified; radiographic evidence of pulmonic disease at the initial presentation was recorded in all five cats that had recurrence of clinical signs attributable to mycobacterial disease, compared to seven cats (54%) that did not redevelop clinical signs of mycobacteriosis. Pulmonic disease was the only factor remaining in the model following multidirectional stepwise reduction. 

## 3. Discussion

This study investigated serial IGRA testing in cats with active mycobacterial disease and whether it can be used to monitor the response to antimycobacterial treatment. Fourteen out of 18 (78%) cats were persistently positive across the pre- and end-of-treatment IGRA, despite apparent clinical resolution of disease. Where comparable, paired OD values for PPDB and E6C10 antigen responses showed no statistically significant difference between the pre- and end-of-treatment IGRA, although the median OD value for both antigens was slightly lower at the end-of-treatment IGRA compared to the pre-treatment results. There was also no statistically significant difference between the pre-treatment OD_PPDB_ value for cats that remained persistently positive to those that reverted to negative at the end-of-treatment IGRA. No statistically significant factors were identified to predict reversion to negativity at the end-of-treatment IGRA, and there was no association between the end-of-treatment IGRA result and recurrence of disease post-treatment. However, all five cats that had recurrence of clinical signs attributable to mycobacteriosis had evidence of pulmonic disease as part of their initial diagnostic investigation, compared to similar changes being identified in only seven of 13 cats (54%) that did not have recurrence of clinical signs during the available period of follow-up. 

In line with previous studies on mycobacterial infections in Great Britain [8], most cases reported here were neutered male cats. The most common breed was the DSH, which reflects the domestic cat population in Great Britain, and the median age was nearly seven years old, although disease was identified in cats as young as six months old. While only seven cats presented with cutaneous lesions, 13 had a lymphadenopathy. Typically, cases of feline mycobacterial disease present with cutaneous nodules, which may be ulcerating and/or have discharging sinus tracts, with a secondary lymphadenopathy [8,24]. It may be the case that small skin lesions were not identified in some of the cats in this study, or subcutaneous masses closely associated with peripheral lymph nodes (i.e., submandibular and popliteal lymph nodes) may have not been identified as unique entities, or these cases presented at a later stage where lymph node changes were more prominent. An abnormal radiographic lung pattern was present in two-thirds of the cats in this study (12 of 18), with a bronchial/interstitial pattern most reported, which is in keeping with previous studies [25,26]. This is putatively due to haematogenous spread of bacteria from the primary site of infection, rather than inhalation of aerosolised mycobacteria which would more likely result in cavitary lesions. Clinical resolution of disease with no evidence of recurrence of clinical signs attributable to mycobacterial disease during the period of follow-up for each individual cat was achieved in 13 cats (72%), which is substantially higher than previously reported [27]. Other than one cat (case 15), all were treated with a combination of rifampicin, a macrolide, or azalide, and a fluoroquinolone for at least two months, although the choice of drugs and the duration varied greatly between cases. While further investigation is required, this would suggest the use of some combination of these drugs is suitable for treating cases of feline mycobacterial disease.

Other than in a small number of cases [9,22], the use of serial IGRA for monitoring the response to antimycobacterial treatment in cats has not been investigated. The data from the current study reflect observations from human studies of treating active mycobacterial disease, in that reversion to IGRA negativity at the end of antimycobacterial therapy is uncommon [19]. Using feline-specific cut-off values to score IGRA antigen responses (Mitchell et al., manuscript in preparation), all cases included in this study were positive at the pre-treatment IGRA, and 78% of cases remained persistently positive at the end-of-treatment IGRA despite apparent clinical resolution. This rate of persistent positivity is similar to a pooled value of persistently positive humans following treatment for active TB (*p* = 0.33); of 982 patients, 660 remained persistently positive (67%) [19]. It has been suggested that maintenance of IGRA positivity could be due to improvement of the immune response in individuals undergoing treatment [28] or an increase in the proportion of IFNγ-secreting CD4+ T-cells [18]. It has also been recognised that clinically healthy individuals previously treated for tuberculosis can retain specific effector memory T-cell responses long after successful resolution of disease, although the precise mechanism driving this remains unknown [29].

Quantitative classification of the IGRA response in animals is less straightforward than in people, as these results are often reported as OD values [30,31]. Intra-subject variability with repeat IGRA testing is recognised, further complicating the ability to accurately compare serial IGRA results [28,32]. This variability can be problematic when interpreting borderline test results, possibly accounting for reversions in the absence of treatment, or conversions with no known exposure to *Mycobacterium* species [33]. In this study, a decrease in the median OD value was observed between the pre- and end-of-treatment IGRA for both PPDB and E6C10, although these differences were not statistically significant. Some cats showed an increase in the OD value between tests, despite apparent clinical resolution; this may be a result from continued expansion of T-cell subsets despite a decrease in the antigenic load [11]. Other studies have shown a decrease in the response to RD-1 antigens to be a better indicator of response to antimycobacterial therapy compared to PPD [15], but this was not observed in this study. This could be due to the small number of cats infected with mycobacteria that encode ESAT-6/CFP-10 in this study, and that approximately 20% of cats infected with *M. bovis* may not produce a substantial IFNγ response to the E6C10 cocktail [9]. 

It has been suggested that IGRA reversion is more likely in humans with a lower pre-treatment IFNγ concentration [13,34]; however, this observation is not consistent [17]. End-of-treatment IGRA reversion was identified in four cats in this study. While the pre-treatment median OD_PPDB_ value for cats that reverted (where comparable data was available) was lower than those that remained positive, this difference was not statistically significant. This is probably influenced by the small study population. Logistic regression did not identify any factors associated with reversion to IGRA negativity; although not significant (*p* = 0.08), this study did show a slight trend with a decrease in the odds of reverting to a negative end-of-treatment IGRA with an increase in age (OR 0.50 [0.23–1.09]). This is similar to studies in humans, where older individuals were more likely to remain positive at the end-of-treatment IGRA despite resolution of disease [35]. One feature of ageing in humans is an increase in the number of memory T-cells; however, chronic antigenic stimulation can also result in the development of an oligoclonal population with impaired function [36]. While there are few studies describing changes in the feline immune system with age, it has been shown that older cats have reduced numbers of many cell populations, including CD4+ T-cells and CD56+ Natural Killer cells, as well as a reduction in the CD4:CD8 ratio [37,38]. Changes in immune cell populations and function with age may be reflected with this decrease in the odds of IGRA reversion with age.

In the current study, recurrence of disease was not associated with a persistently positive pattern of IGRA results, albeit the number of cats with recurrence of disease was low so the results should be interpreted cautiously. All cats who re-presented with clinical signs suggestive of mycobacterial disease had evidence of pulmonic disease on thoracic imaging when they initially presented. This may reflect a reduced capacity for the host immune system to restrict the spread of mycobacteria from the primary site of infection, or differences in the virulence of different species and/or strains of mycobacteria. Consequently, once treatment has been stopped on the basis of the cat achieving clinical resolution, any mycobacteria that may have been dormant or hidden from the immune system could reactivate, resulting in the recurrence of clinical signs associated with mycobacteriosis. Previous studies have shown that while both radiography and CT are of benefit in the investigation of cases of feline mycobacteriosis [25,26], CT is more sensitive at detecting pulmonic changes attributed to mycobacterial infection, and that post-treatment small lesions may be missed with conventional radiography [22]. In the current study, most cats underwent radiography rather than CT, which may have not identified small regions of lung pathology at the end of treatment, resulting in treatment being withdrawn early and subsequent relapse. Data from cattle experimentally infected with *M. bovis* and treated with isoniazid showed an increase in the IFNγ response to stimulation with ESAT-6/CFP-10 after treatment was stopped, most notably in cattle with visible lesions at post-mortem examination, indicating incomplete treatment and failure to eliminate all mycobacteria [39]. One complicating factor is the dynamics of feline mycobacterial infections; although four of the five cats re-presented with clinical signs identical to their initial presentation, this could be due to reinfection from environmental sources, i.e., hunting prey, or true recrudescence of disease. Further analysis of treatment outcomes in cases of mycobacterial disease is needed. Altogether, these results suggest that attaining IGRA negativity in cats treated for mycobacterial disease is unlikely, and it does not imply any benefit regarding long-term outcomes.

There are limitations to this study, most notably the small sample size. While there were 594 individual cats that had been tested by IGRA, only 107 of these had more than one IGRA performed, and of these only 18 met the final inclusion criteria. Cats with a previous history of mycobacterial disease were excluded, as it has been shown in humans that individuals with a history of TB later presenting with non-TB disease (including bronchopneumonia and lung neoplasia) can still generate a positive IGRA result that cannot be distinguished from those with active TB [40]. Additionally, these were all naturally occurring cases that were managed in practice by the referring veterinary surgeon (RVS), with the authors overseeing case treatment remotely. Since the authors often became involved after the initial treatment choices had been made, there was no standardised approach; this was exacerbated by recent changes in treatment guidelines [2,41]. A prospective study with a defined cohort of patients, tested at set time points, would provide stronger conclusions; however, the current study provides ‘real world’ representations of clinical data, management of cases, and the limitations of test failures. 

To conclude, serial IGRA testing to monitor the response to antimycobacterial chemotherapy is of limited benefit in the cat; however, it may be warranted to perform an end-of-treatment IGRA. Provided there is reversion to negativity, if the cat were to re-present with suspected relapse or reinfection than an IGRA could be repeated, with a positive result suggesting active mycobacterial disease. However, if the end-of-treatment IGRA were to remain positive, any further presentation with signs compatible with mycobacteriosis would require demonstration of Ziehl–Neelsen (ZN)-positive organisms. If these cannot be identified, alternative testing modalities, such as PCR or culture, would be advisable.

## 4. Materials and Methods

### 4.1. Study Population Selection

A retrospective analysis of IGRA results from cats tested for mycobacterial disease by Biobest Laboratories, Scotland, between May 2013 and October 2019 was undertaken. Each assay had been performed as previously described [9,31]. Test submission data and results were cross-referenced with clinical data supplied by the RVS to the authors for case advice and management. This was done in line with owner consent, and the RVS was contacted for further clinical information when necessary. Individual cats with multiple IGRA results were identified. The IGRA OD values, a correlate for the amount of IFNγ secreted by T-lymphocytes in response to antigenic stimulation [30], were re-assessed in accordance with current guidelines [42], which have subsequently been adapted for feline-specific thresholds (Mitchell et al., manuscript in preparation). Briefly, for a test to pass QC thresholds, the mean OD_PC_ had to be ≥ 0.40 and exceed the OD_NC_ by at least 0.10 OD units i.e., OD_PC_-OD_NC_ ≥ 0.10; if the OD_PC_ was < 0.40 it was considered a borderline pass provided OD_PC_-OD_NC_ ≥ 0.20. The OD_NC_ also had to be ≤ 0.30. The CV for duplicate control and test well OD values had to be within 30%. Individual cats without at least two tests that had passed these criteria were excluded.

Supporting clinical data was consulted for each cat (where available) to identify those with a final diagnosis of mycobacterial disease, either (1) confirmed with culture or PCR, or (2) strongly suspected (i.e., supportive histopathology and/or ZN-positive staining of tissue biopsy material, but no culture of PCR). Cats were excluded if they had insufficient clinical records or had been tested as potential in-contact cases. Cats were also excluded if they had been previously diagnosed and treated for an incidence of mycobacterial disease prior to IGRA testing. To be included in this study, all cats had to have an IGRA performed at the point of starting treatment for mycobacterial disease, and at the point of clinical resolution of active disease when treatment was stopped. 

### 4.2. Qualitative Interpretation of Results

For each cat that met the inclusion criteria described above, IGRA results were assessed to identify a positive response to any of the test antigens. For antigen positivity, the OD value for cells stimulated with PPD from *M. avium* (PPDA) and PPD from *M. bovis* (PPDB) had to exceed the OD_NC_ by 0.07, whereas the OD value for the antigenic cocktail ESAT-6/CFP-10 had to exceed the OD_NC_ by 0.05. A PPDA-biased response was given as OD_PPDA_-OD_PPDB_ ≥ 0.05, and a PPDB-biased response as OD_PPDB_-OD_PPDA_ ≥ 0.05 [43]. A test showing a positive response to any antigen was called positive, whereas a test lacking a positive response to all antigens was called negative. Clinical records were consulted to correlate the timing of IGRA tests with treatment. An IGRA was termed pre-treatment if it was performed within two weeks of starting treatment for mycobacterial disease, or before treatment had been instigated. The IGRA was deemed end-of-treatment if treatment had been stopped within the proceeding two weeks, or if treatment was ceased within four weeks of the test being performed. Once assigned as positive or negative, the pattern of IGRA results was interpreted as follows: those that remained positive across both tests were classified as persistently positive; those that changed from positive to negative were classed as reversions. A one-sample chi-squared test was performed to determine if the number of cats with a negative end-of-treatment IGRA differed from a hypothesised proportion of reversion of 0.33 in humans with active TB [19]. Statistical significance was set to p < 0.05, and data were analysed using RStudio Ver 1.2.1335 (RStudio, Inc., Boston, MA, USA) [44]. 

### 4.3. Quantitative Interpretation of Results

Negative control-corrected paired OD_PC_ values were compared across both testing points for each cat by plotting the average OD_PC_ value for each test with a 95% confidence interval for the mean; tests with overlapping confidence intervals were considered comparable. Wilcoxon signed-rank tests were performed to compare pre- and end-of-treatment IGRA OD_PPD_ and OD_E6C10_ values. The selection of either PPD antigen of OD_PPD_ analysis was based on demonstration of PPD-bias. Comparison of OD_E6C10_ values was undertaken for cats who were IGRA-positive for this condition or were diagnosed by culture or PCR with *M. bovis*, which encodes the genes for this antigenic cocktail (unlike *M. microti*). To investigate whether cats who reverted from positive to negative at the end-of-treatment IGRA had a lower pre-treatment OD_PPD_ value compared to those who remained positive, a Mann-Whitney U test was performed. Statistical significance was set to *p* < 0.05. Data were analysed using RStudio Ver 1.2.1335, and graphs were created using GraphPad Prism Ver 9.0.0 (GraphPad Software, San Diego, CA, USA). 

### 4.4. Identification of Factors Associated with IGRA Reversion and Recurrence of Disease

For cats with a valid pre- and end-of-treatment IGRA, logistic regression was performed to identify predictors of reversion to negativity at the end-of-treatment IGRA, and of disease recurrence after the cessation of antibiotic therapy. A multivariate model with all potential explanatory factors was constructed for each outcome (reversion and recurrence). A multidirectional stepwise approach was used to reduce the multivariate model to its simplest components. Statistical significance in the reduced model was set to *p* < 0.05. Logistic regression was performed using RStudio Ver 1.2.1335. 

## Figures and Tables

**Table 1:** Summary of the details of the cats included in this study.

Case	Age (Years)	Gender	Breed	Culture/PCR	FeLV Ag/FIV Ab	Serum Calcium Concentration	Clinical Disease	Treatment & Duration (Months)	Outcome & Follow-up Duration (Months)
1	7	MN	DSH	MTBC ^a^	NP	NP	Retrobulbar ocular mass, submandibular lymphadenopathy	R/A/P (3); surgery	Resolved (9)
2	3	FN	Siamese	*M. bovis* ^b^	NP	NP	Lip mass, submandibular lymphadenopathy, bronchointerstitial lung pattern ^c^	R/A/P (6)	Resolved (18)
3	0.5	MN	DSH	MTBC ^a^	NP	Normal (total)	Interstitial lung pattern ^c^	R/A/P (3); R/P (15)	Resolved (3)
4	8.5	MN	DSH	NP	Negative	Increased (total) Normal (ionised)	Nasal mass, submandibular lymphadenopathy	R/A/P (6)	Resolved (48)
5	7	MN	Siamese	*M. microti* ^a,b^	Negative	Normal (total)	Multiple cutaneous masses, bronchointerstitial lung pattern ^c^	R/A/P (2); R/P (6)	Resolved (12)
6	7	MN	DSH	NP	NP	NP	Bilateral submandibular lymphadenopathy, bronchointerstitial lung pattern ^c^	R/A/P (7)	Recurrence of submandibular lymphadenopathy 27 months later; retreated with R/A/P, lost to follow-up
7	6	MN	Siamese	MTBC ^a^	NP	NP	Submandibular mass, submandibular lymphadenopathy, bronchial lung pattern ^c^	R/A/P (3)	Re-presented with mass on lip 17 months later; retreated with R/A/P for four months, resolved
8	11	MN	Siamese	NP	Negative	Increased (ionised)	Tonsillar lymphoid hyperplasia, alveolar lung pattern ^c^	R/A/M (3); A/M (12)	Resolved (12)
9	7	MN	DSH	*M. bovis* ^b^	NP	NP	Conjunctival mass	R/A/P (3); A/P (2)	Resolved (6)
10	6.5	FN	DSH	Negative ^b^	NP	NP	Multiple cutaneous masses, peripheral lymphadenopathy bronchointerstitial lung pattern ^c^	R/A/P (8)	Recurrence of cutaneous masses and peripheral lymphadenopathy eight months later; euthanased
11	5	MN	DSH	*M. bovis* ^b^	NP	Normal (total)	Discharging cutaneous mass, perihilar lymphadenopathy, interstitial lung pattern ^c^	R/A/P (6); A/P (18)	Resolved (42)
12	13	MN	Bengal	NP	NP	Normal (total)	Generalised lymphadenopathy, bronchointerstitial lung pattern ^d^	R/A/P (9)	Recurrence of clinical signs three months later; euthanased
13	5.5	MN	DSH	Negative ^b^	NP	NP	Generalised lymphadenopathy, bronchointerstitial lung pattern ^c^	R/A/P (6)	Resolved (6)
14	7.5	MN	DSH	NP	Negative	NP	Multiple cutaneous masses, submandibular lymphadenopathy	R/P/Cd (3); R/A/P (3)	Resolved (36)
15	3	FN	DSH	NP	NP	NP	Submandibular lymphadenopathy	E/D/P (6)	Resolved (6)
16	1.5	MN	Tonkinese	NP	Negative	Increased (total, ionised)	Diffuse interstitial lung pattern ^c^	R/Ct/P (8)	Recurrence of clinical signs 16 months later; retreated with R/Ct/P for eight months, resolved
17	8.5	MN	DSH	NP	NP	Normal (total)	Generalised lymphadenopathy	R/E/P (4)	Resolved (24)
18	2	MN	DSH	Negative ^a,b^	NP	Increased (total)	Panuveitis, bronchointerstitial lung pattern ^d^	R/A/P (3); surgery	Resolved (12)

FeLV Ag = feline leukaemia virus antigen. FIV Ab = feline immunodeficiency virus antibody. MN = male neutered. FN = female neutered. DSH = domestic short-hair. MTBC = *Mycobacterium tuberculosis*-complex. NP = not performed. R = rifampicin. A = azithromycin. P = pradofloxacin. M = marbofloxacin. E = erythromycin. D = doxycycline. Ct = clarithromycin. Cd = clindamycin. ^a^ = PCR diagnosis. ^b^ = culture diagnosis. ^c^ = thoracic radiography. ^d^ = computed tomography.

**Table 2:** Binary classification and interpretation of interferon-gamma release assay (IGRA) results prior to starting antimycobacterial therapy and at the point of treatment cessation, and categorisation of the pattern. Positivity was ascribed to an IGRA with a positive result to any test antigen, whereas a negative result indicated an optical density value below positivity-thresholds for all test antigens.

Case	Pre-Treatment IGRA	IGRA Result	End-of-Treatment IGRA	IGRA Result	Pattern
1	Positive	B > A	Positive	B > A	Persistent positive
2	Positive	B > A	Positive	B > A	Persistent positive
3	Positive	B > A	Positive	B > A, E positive	Persistent positive
4	Positive	B > A	Positive	B > A	Persistent positive
5	Positive	B > A	Positive	B > A	Persistent positive
6	Positive	B > A	Positive	B > A	Persistent positive
7	Positive	B > A	Positive	B > A	Persistent positive
8	Positive	B > A	Positive	B > A, E positive	Persistent positive
9	Positive	B > A, E positive	Positive	B > A, E positive	Persistent positive
10	Positive	B > A	Positive	B > A	Persistent positive
11	Positive	B > A, E positive	Positive	B > A, E positive	Persistent positive
12	Positive	B > A	Positive	B > A	Persistent positive
13	Positive	B > A	Positive	B > A	Persistent positive
14	Positive	B > A	Positive	B > A	Persistent positive
15	Positive	B > A	Negative	Negative	Reversion
16	Positive	B > A	Negative	Negative	Reversion
17	Positive	B > A	Negative	Negative	Reversion
18	Positive	B > A, E positive	Negative	Negative	Reversion

Tuberculin-bias is indicated by >. B = purified protein derivative (PPD) from *M. bovis* (PPDB). A = PPD from *M. avium* (PPDA). E = early secreted antigenic target 6kDa/culture filtrate protein 10kDa (ESAT6/CFP10).

## Data Availability

The data presented here are available on request from the corresponding author. The data are not publicly available due to privacy regarding owner personal information.
